# Complete genome sequence of *Brachymonas denitrificans* KE5, an aromatic compound–degrading bacterium isolated from livestock wastewater

**DOI:** 10.1128/mra.00940-26

**Published:** 2026-10-14

**Authors:** Kook-Il Han, Young Ho Nam, Byung-Gon Ryu, Young Taek Oh

**Affiliations:** 1 Nakdonggang National Institute of Biological Resources (NNIBR) 443035, Sangju-si, Gyeongsangbuk-do, Republic of Korea; Rochester Institute of Technology, Rochester, New York, USA

**Keywords:** *Brachymonas denitrificans* KE5, genome sequence

## Abstract

We report the complete genome sequence of *Brachymonas denitrificans* KE5, isolated from livestock wastewater. Genome analysis revealed genes involved in aromatic compound degradation, including dioxygenases and catechol degradation pathways, indicating their potential for biodegradation of organic pollutants.

## ANNOUNCEMENT

*Brachymonas denitrificans* is a gram-negative, aerobic bacterium belonging to the family Comamonadaceae (1).

We present the complete genome sequence of *B. denitrificans* strain KE5, isolated in August 2024 from swine wastewater in Sangju, Gyeongsangbuk-do, Republic of Korea. To enrich the target microorganisms, 5 g of the slurry sample was added to minimal salt medium (prepared in-house using reagents from Sigma-Aldrich, USA, as previously described [2]) and incubated aerobically for 14 days at 30°C and 150 rpm. Ten-fold serial dilutions (10^−1^ to 10^−10^) were prepared using sterile 0.85% (wt/vol) NaCl solution and plated on tryptic soy agar (TSA; BD Biosciences, USA) plates, which were kept at 30°C for 48 h. Subculturing yielded a pure single colony.

For whole-genome sequencing, strain KE5 was cultured on TSA at 30°C for 3 days, followed by genomic DNA extraction using the Maxwell RSC Tissue DNA Kit (Promega, USA) according to the manufacturer’s protocol without modifications. After shearing the DNA with a g-TUBE (Covaris, USA), long-read libraries were generated with the SMRTbell Express Kit (PacBio). DNA fragments under 15 kb were removed via size selection on a BluePippin system (Sage Science, USA). Sequencing was performed on a PacBio Revio platform, and raw reads were adapter-trimmed and quality-filtered using SMRT Link v25.1 (3). Short-read sequencing libraries were prepared with the TruSeq Nano DNA Kit (Illumina, USA) and sequenced on the Illumina NovaSeq 6000 platform. Raw short reads were trimmed using Trimmomatic v0.38 (4). *De novo* genome assembly of long reads was performed using Canu v2.2 (5) within SMRT Link v25.1 (3). The assembled genome was refined through two iterations of polishing with Pilon v1.22 (6) using Illumina short reads, followed by coverage analysis via BWA-MEM v0.7.17 (7).

**Fig 1:**
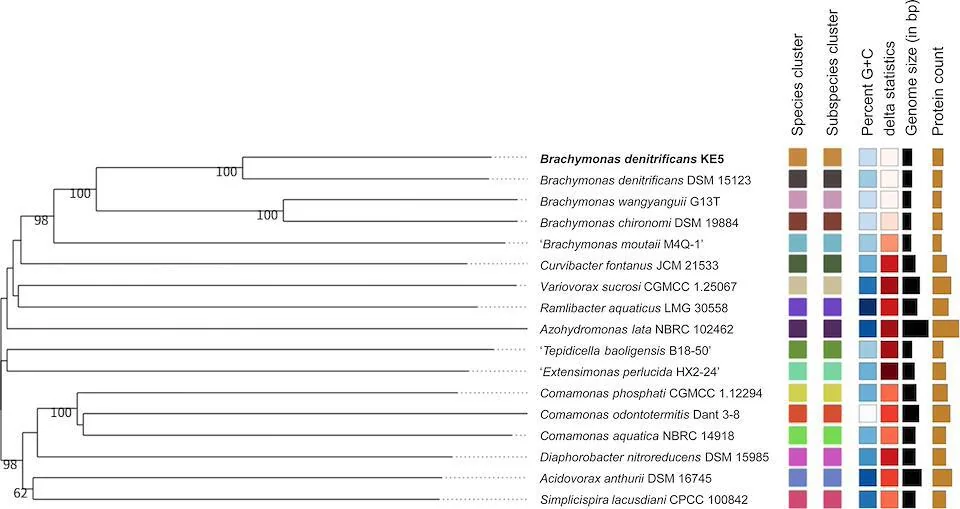
Whole-genome phylogenetic tree of strain KE5 and related type strains generated using the Type Strain Genome Server. The phylogenomic tree was inferred with FastME 2.1.6.1 (12) based on Genome BLAST Distance Phylogeny distances calculated using formula *d*₅. Bootstrap support values (>60% from 100 replicates; overall mean support of 79.6%) are indicated above nodes, and the tree was midpoint-rooted (13). Strain KE5 exhibited the highest genomic affinity to *Brachymonas denitrificans*. Leaf labels denote species/subspecies cluster assignments, G + C content (%), delta values, genome size (bp), and total protein counts (from left to right).

Sequencing generated 44,829 PacBio reads (N50, 6,708 bp; 273.2 Mb) and 23,620,488 Illumina paired-end short reads (2 × 150 bp, totaling 3.6 Gb). Evaluation of read depth and replicon circularization confirmed assembly quality, achieving a mean genome coverage of 97.06×. Genome annotation was conducted with the National Center for Biotechnology Information Prokaryotic Genome Annotation Pipeline v6.11 using GeneMarkS-2+ (8). Default parameters were used except where otherwise noted. Digital DNA-DNA hybridization (dDDH) analysis (GGDC 4.0 [9, 10]) showed 75.6% dDDH with the type genome of *B. denitrificans* DSM 15123 (GenBank accession: GCA_900110225). The complete chromosome is 2,814,929 bp with a G + C content of 64.0%, containing 2,664 genes (2,571 protein-coding genes). BUSCO v5.1.3 (11) analysis indicated 98.39% completeness (0.81% duplicated). The Type Strain Genome Server-based midpoint-rooted phylogenomic tree (12, 13) revealed that strain KE5 clustered closely with the type strain *B. denitrificans* DSM 15123 (Fig. 1).

Strain KE5 possesses genes encoding putative proteins involved in aromatic ring degradation (*catA*, *pcaH*, and *pcaG*), aliphatic compound degradation (*alkB*), dehalogenation (*had*), heavy metal resistance (*arsC*, *arsR*, and *ctpA*/*copA*), toxic efflux systems (*mexD*/*cusA*), and oxidative detoxification (CYP family) (Table 1), underpinning bioremediation potential for odor-causing compounds.

**TABLE 1:** Assembly statistics and genomic features of *B. denitrificans* KE5 from livestock wastewater in the Republic of Korea

Parameter	Result for *B*. *denitrificans*
Sample source	Livestock wastewater
Sampling location	Swine wastewater treatment plant, Sangju-si, Republic of Korea
Sample type	Manure
Year of isolation	2022
No. of reads	PacBio (44,827), Illumina (23,620,488)
Type of reads	Paired end
Genome length (bp)	2,814,929
No. of contigs	1
GC content (%)	64.0
Sequencing depth (×)	97.06
Coding DNA sequences	2,571
tRNAs	54
ncRNA	3
rRNAs	3, 3, 3 (5S, 16S, 23S)
Biodegradation genes	10 (*catA*, *pcaH*, *pcaG*, *alkB*, *had*, *arsC*, *arsR*, *ctpA/copA*, *mexD/cusA*, *CYP*)
CRISPR array	3
Accession no.	JBYLRO010000000

## Data Availability

The genome sequence and raw reads for strain KE5 were deposited in GenBank under accession number JBYLRO010000000, BioProject PRJNA1399525, BioSample SAMN54450835, and Sequence Read Archive SRX31787929.
